# Complementing global chemicals management through shaping consumer behavior

**DOI:** 10.1016/j.isci.2025.112700

**Published:** 2025-05-19

**Authors:** Brij Mohan Sharma, Jane Muncke, Justin M. Boucher, Lisa Zimmermann, Thomas A. Brunner, Poonam Arora, Martin Scheringer

**Affiliations:** 1 RECETOX, Faculty of Science, Masaryk University, Kotlarska 2, Brno, Czech Republic; 2 Institute of Biogeochemistry and Pollutant Dynamics (IBP), ETH Zürich, 8092 Zürich, Switzerland; 3 Food Packaging Forum (FPF) Foundation, 8045 Zürich, Switzerland; 4 Food Science and Management, School of Agricultural, Forest and Food Sciences (HAFL), Bern University of Applied Sciences (BFH), 3052 Zollikofen, Switzerland; 5 School of Business, Quinnipiac University, Hamden, CT 06518, USA

**Keywords:** Industrial chemical, Engineering, Social sciences

## Abstract

The rapid expansion of the global chemical industry, fueled by consumerism and economic growth, has created severe environmental and public health challenges. The current chemicals management approach primarily regulates the “production system”, setting standards and imposing large responsibilities on the chemical industry. However, this approach has been found inadequate as it often neglects the vital role of the “consumption system” in driving chemical production and use, and pollution caused by chemicals. To address this imbalance, we propose a systematic integration of behavior-shaping tools into the global and local chemical management strategies, aimed at shifting consumer behavior toward safer and more sustainable chemical consumption. By applying ethical marketing and social- and behavioral-science techniques, consumers, including risk-sensitive groups such as women of childbearing age and children, can be nudged and empowered to make and adopt safer and mindful chemical choices, ultimately reducing their exposure to toxic chemicals. This consumer-oriented approach complements traditional “industry-focused” chemical regulations. Such an integrated approach (with management roles spanning across different stakeholders) is particularly required in regions with outdated or weak regulatory enforcement. Furthermore, fostering consumer demand for safer and more sustainable chemicals consumption will incentivize chemical industry innovations and encourage the market to move toward safer alternatives. Ultimately, a comprehensive integrated approach that focuses on both production and consumption systems could better strengthen global chemicals management, leading to improved environmental and public health outcomes and advancing progress toward the Sustainable Development Goals.

## Why: Current ineffectiveness of chemical regulations

Chemical pollution has been identified as one of the nine planetary boundary threats to the environment and human life on Earth.^1^ Together with climate change and biodiversity loss, chemical pollution poses as a major hurdle in achieving the United Nations Sustainable Development Goals (SDGs) and preserving the Earth’s finite capacity to sustain the well-being of all lives.^2^^,^^3^ A recent study by the World Health Organization (WHO) estimated that exposure to only a small subset of toxic chemicals (for which exposure and risk data are available) contributed to about 2 million deaths globally in 2019. These deaths were directly linked to chemicals-associated health impacts, including poisonings, cardiovascular diseases, chronic respiratory diseases, and cancers.^4^ Furthermore, health damage from chemical pollution not only contributes to mortality and morbidity, but also costs economically. For instance, lead exposure alone cost USD 6 trillion globally in 2019 (equivalent to 6.9% of the global gross domestic product (GDP)),^5^ and just a handful of the many hazardous chemicals in plastic materials were estimated to cost the United States about USD 250 billion in disease burden in 2018.^6^ Both health consequences and economic costs of chemical pollution are expected to be more severe in developing countries, where pollution is dire, progress to managing pollution sources is slow, and is restrained by other pressing environmental, health, and economic challenges including air pollution, vector-borne and pathogenic diseases, and a high-risk economic landscape.

To manage chemical pollution and protect human health and the environment, world leaders, scientists, citizens, and civil society representatives have strongly relied upon regulatory frameworks for decades. Typically, these regulations follow retrospective approaches to managing chemical pollution and its effects, implying that only chemicals historically known to be harmful to humans and the environment are likely to be banned or restricted.^7^ In addition, they are often regarded as a panacea for managing a wide variety of chemicals–differing in origin, physicochemical properties, and toxicity– across the highly dynamic socioeconomic and political spectrums existing across many regions. In recent years, chemical regulations in some regions, such as the European Union (EU), have undergone significant transformations. In particular, prospective management approaches have been adopted, placing greater emphasis and responsibility on chemical manufacturers to identify and manage the risks linked with the chemicals they produce and market within the EU.^8^

At the same time, considering the global chemical predicament, researchers and think tanks have started to acknowledge the inefficacy and failures of global, regional, as well as national chemical regulations.^9^^,^^10^^,^^11^ Furthermore, recent research raises serious concerns regarding the effectiveness of well-recognized multilateral agreements managing a wide range of toxic chemicals including those banned under multilateral environmental agreements (MEAs) like the Stockholm Convention (SC).^12^^,^^13^^,^^14^ For instance, despite decades of ban on the use, production, and trade of polychlorinated biphenyls (PCBs), the estimated generation of PCBs in the United States peaked in 2019 (∼40 million kg) due to the emergence of by-product PCBs.^15^ Managing synthetic toxic organic chemicals, such as PCBs and other persistent organic pollutants (POPs), is particularly challenging. One reason is their complex physicochemical properties, especially in terms of their persistence and bioaccumulation, which often lead to disagreements over their risk classifications and criteria.^16^^,^^17^ Other reasons include their widespread industrial use, illegal trade, and challenges in identifying their release, exposure sources, and alternatives.

Even more concerning is the fact that regulations have not been fully effective in protecting human health and the environment from relatively “unsophisticated” toxic chemicals, such as lead and mercury. These chemicals have comparatively simpler physicochemical properties and are well-understood in terms of their release, exposure sources, and health effects.^18^^,^^19^ Yet, their use, release, and exposure continue to pose significant challenges for global chemicals management.^20^^,^^21^ This issue is particularly prominent in developing countries, where the scope of the problem is poorly acknowledged and underreported, existing local regulatory frameworks are outdated and less robust, and the issue receives inadequate attention from chemical regulatory authorities.^22^

An important factor affecting the overall efficacy and coverage of existing chemical regulations is the extraordinary size of the global chemical industry, which accounts for about 7% of the global GDP. Research shows that only a small fraction of the vast number of chemicals currently circulating in the global market have ever been analyzed for their presence in the environment.^23^^,^^24^ Scientific data on the toxicity of most chemicals currently present in the market are either inadequate or completely missing. For instance, of the over 16,000 chemicals known to be present in plastic materials and products, over 10,000 have no data on their hazards and toxicity.^25^ Considering the vastness of the chemical industry, estimates suggest that it may take thousands of years to evaluate all existing synthetic chemicals for their human and environmental safety.^26^^,^^27^^,^^28^ This challenge is further exacerbated by uncertainties, lack of transparency, and variations in toxicity and risk assessment methodologies.^29^^,^^30^^,^^31^^,^^32^ For instance, exposure limits used to assess environmental risks can vary by as much as five orders of magnitude, even when derived using the same framework.^33^^,^^34^ Such pronounced variability introduces considerable uncertainty, impacting regulatory frameworks and management decisions.^34^ The case of Bisphenol A (BPA), where the very definition of BPA as an endocrine disruptor remains contested, presents a classic example of how disparities in toxicity and risk assessment methods and outcomes can shape the development and implementation of chemical regulations. This is particularly the case in current circumstances where industry uses such disparities in scientific assessments to dilute regulatory efforts on chemical safety of consumer products to maximize their profits.^35^^,^^36^

An additional concern is that the effectiveness of global chemical regulations faces significant obstacles due to disparities between developed and developing countries on various fronts. These disparities include differences in the robustness and scope of existing national and regional chemical regulation, variations in mechanisms of their implementation, and economic priorities. Furthermore, local and regional geopolitical dynamics play an important role in chemicals management,^37^^,^^38^ as does the uneven representation of scientific and political leaderships at both local and international platforms.^18^ Due to these disparities, chemical regulations often fail to fully achieve their intended objectives “uniformly” and “timely”, irrespective of their comprehensiveness. Several examples from around the globe illustrate this issue. For instance, India, despite having relatively modern regulations on waste and air quality management, has severely struggled for decades to effectively address these challenges.^39^^,^^40^ Its capital Delhi, which is also home to the headquarters and regional centers of various national and international environmental and chemical regulatory agencies, vividly demonstrates this struggle. Here, millions of vulnerable individuals, including newborn children and women of childbearing age, are left exposed to toxic chemicals with detrimental health consequences from unmanaged wastes and air pollution.^41^ In contrast to developed countries, the effectiveness of overall environmental (including chemical pollution) regulations in many developing countries is hindered by the short-term vision of ruling political parties, which leads to poor chemical management infrastructure and planning.^42^ Additionally, the centralized nature of governance, combined with limited decentralization of power and actions at local levels,^43^ along with a dire lack of public awareness and participation, further exacerbate the problem.

An additional layer of complexity in the landscape of global regulations on chemicals is added by the inequality of purchasing power, which is manifest in consumerism and market dynamics. Price disparities in different markets often result in hazardous chemical products being proportionately supplied and sold to marginalized communities, both domestically and internationally.^44^^,^^45^ These communities, which also comprise lower-income and historically oppressed ethnic groups, bear the brunt of exposure to substandard or harmful products. For instance, inexpensive and poorly regulated cosmetic products, such as skin-lightening creams containing toxic chemicals including mercury, are often marketed to economically disadvantaged populations through manipulative advertisements or by being made easily accessible.^46^^,^^47^ Such systematic patterns highlight how social and economic inequalities compound the challenges of equitable and effective chemicals management.

Regulatory disparities between developed and developing countries, both in terms of structure and effectiveness of chemical regulations, lead to a cascade of interconnected chemical management issues. These disparities can exacerbate overall environmental management challenges and perpetuate socioeconomic unfairness in this context.^48^ Resulting issues from this imbalance include illegal chemical use and trade, exploitation of economically vulnerable countries and communities within them, and the relocation or recreation of pollution hotspots to new areas in pristine regions.^13^ As an example, thousands of tonnes of banned pesticides were exported from the UK and EU to developing countries worldwide in 2018.^49^ Such activities can contribute to the creation of new chemical pollution hotspots in developing countries that already lack robust regulations and economic resources to address their existing chemical pollution issues.^50^

A key concern with current global regulations on chemicals management is their limited success in addressing the production and use of toxic and unsafe chemicals, even in regions with modern regulatory frameworks, such as the EU.^51^ Implementing regulations to oversee the chemical industry has faced numerous challenges worldwide. One primary reason is that industries often use political and economic influence to push for less stringent regulations that align with their business interests.^52^ This was evident recently when per- and polyfluoroalkyl substances (PFAS) management actions in the U.S. and EU were moderated, despite strong proposals and demands from scientists and civil society organizations (CSOs). In fact, the largest current and former PFAS manufacturers spent about $55.7 million between 2019 and 2022 lobbying on PFASs and other chemical regulatory issues.^53^^,^^54^^,^^55^

When chemicals management is approached by merely tapping the chemical industry, i.e., the “production system”, the development and enforcement of regulations are often impacted by a lack of transparency in industry-driven research into the risk and safety of chemicals. Here, “industry” refers to both primary chemical producers and downstream enterprises (secondary producers) using these chemicals for manufacturing articles, consumer products including cosmetics, as well as toys and utensils for children. A notable example of this lack of transparency in industry-driven research is the case of two well-known PFASs, PFOS and PFOA. Their toxicity and health impacts were known, but concealed by the manufacturer long before the public health community and authorities became aware.^56^ The industry’s transparency in fairly disclosing the risks of its chemicals and products, and thereby success in regulatory compliance, can be influenced, to an extent, by the costs of reformulating products and modifying processes when the chemicals and products pose risks to human health or the environment. The cost of regulatory compliance could be significant. For instance, a recent estimate suggests that the cumulative cost for six selected subsectors of the EU chemical industry to comply with certain EU chemical legislation was €2.8 billion during 2004–2014.^57^ These additional costs of regulatory compliance may impact producers’ market performance,^58^^,^^59^ potentially influencing their transparency in self-reporting of their chemical research. For instance, recent developments among the world’s largest chemical producers, such as BASF, demonstrate this trend, with the company no longer publishing phase-out plans for hazardous chemicals.^58^ In many instances, the chemical industry uses the high costs of regulatory compliance to shift attention away from health and environmental impacts of chemicals or to justify attempts to circumvent regulations and their delayed implementation.

Though conventional international strategies, multilateral agreements, and multi-stakeholder collaborations have existed for global chemicals management for the past several decades, additional approaches are still necessary as the existing frameworks continue to exhibit implementation gaps, weak enforcement, and growing fragmentation in global-to-local governance. Many conventional chemicals management initiatives suffer from weak coherence and coordination, with overlapping mandates and inconsistent goals that hinder effectiveness. For example, PCBs are regulated under the SC (governing their use and production) and the Basel Convention (regulating their transboundary movement and disposal of waste containing them), and additionally addressed by SAICM and the Global Framework on Chemicals (GFC) through broader capacity-building efforts.^60^^,^^61^ Despite these overlapping mandates and initiatives, progress in PCB elimination has been slow and inconsistent.^62^ Recent research highlights that more than 10 million tonnes of PCBs remain, even after over 30 years since their production had been banned.^63^ Many countries around the world still have large undocumented stocks of PCB-containing equipment (e.g., old transformers) and lack the infrastructure, capacity, or regulatory mechanisms necessary for their safe collection and disposal by the 2028’s PCB-elimination deadline set by the SC.^63^^,^^64^^,^^65^ Furthermore, the growing complexity and interconnectedness of modern global challenges, including climate change, biodiversity loss, and widespread overall environmental pollution, demand more inclusive and adaptive responses in addition to what many traditional regulatory systems offer.

Regulations undeniably play a crucial role in the systematic management of chemicals and the pollution they cause. However, unlike conventional legal or compliance-related offenses, chemical pollution is increasingly recognized, not just a result of isolated incidents of rule-breaking but as an outcome of a broader complex systematic failure driven by political, economic, and social structural flaws. The usual reactive, linear, and centralized nature of traditional chemical governance is often associated with regional disparities, inhomogeneous coverage and applicability, and vulnerability to industry influence. These limitations in the current approach of chemical governance underscore the immediate need for more effective complementary strategies based on participatory, inclusive, and polycentric governance models.

## What: A necessary look at the consumption system

**Figure 1:**
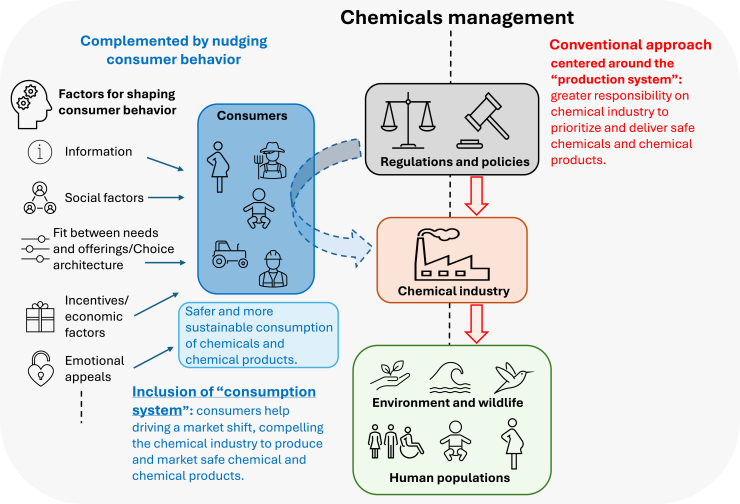
Illustrating how behavioral shaping can complement chemical regulations, for instance, by influencing consumer behavior toward safe and more sustainable consumption, thereby minimizing exposure to hazardous chemicals in consumer products, and by driving market demand for safer products, thereby encouraging industry to better comply with chemical regulations, and manufacture and market safer and environmentally friendly products While this perspective outlines a strategy for influencing consumer behavior and thereby shifting the chemical market dynamics toward safer products, it does not imply that consumers bear primary responsibility for improving overall chemical management. Rather, this approach intends to complement systematic changes driven by regulations and industry accountability.

The challenges discussed in the previous sub-section illustrate that the current regulatory approach to managing chemicals is insufficient, as it primarily focuses on transforming the “production system” of chemicals and chemical products rather than equally addressing the “consumption system”. The limitations of this production-centric approach in managing and mitigating environmental and human health impacts of chemicals have been observed in both developed and developing countries.^66^ To address this gap, we propose a novel perspective that emphasizes the need for modern chemical regulations to systematically include interventions aimed at transforming the “consumption system” and not just the production system. Specifically, to include and transform the chemicals consumption system, we advocate leveraging behavioral nudging as a measure toward safer and more sustainable consumption of chemicals and chemical products (Figure 1).

Behavioral nudging, whether integrated into specific regulatory frameworks or implemented as standalone interventions, is expected to play a crucial role in shaping consumer behavior toward safer and more sustainable consumption of chemicals. At the same time, it enables consumer choices that support a circular economy for chemicals and chemical products.^67^ By emphasizing consumer-focused measures, including behavioral nudging, regulatory bodies can better respond to industrial influences on regulations and increase the robustness of enforcement mechanisms. When regulations are complemented with specific interventions shaping consumer behavior, rather than relying solely on transforming the production system, a niche will emerge for a competitive market driven by increased demand for safe chemicals and chemical products.^68^ This market dynamic will eventually encourage industries to comply with chemical regulations and innovate toward safer and more environmentally friendly chemical products. This market shift will enhance protection for both consumers and the ecosystem alike. Recent studies indicate that consumer choices have led to gradual transformations in the food, beverage, and agriculture (FB&A) industry. For example, recent survey studies suggest that there is an increasing demand for safer chemicals from consumers.^69^ In fact, 60% of the companies studied in recent research expressed a desire to align with consumer demands for safe and sustainable consumption.^70^^,^^71^^,^^72^

## How: The novelty of the behavior nudging concept

Industries have employed behavioral nudging for decades to shape consumer choices and boost their businesses.^73^ These interventions usually enhance industries’ strategic decision-making, improve advertising effectiveness, and mitigate risks associated with large investments.^74^^,^^75^^,^^76^ For example, despite the well-known serious health risks, the tobacco industry utilizes various behavioral nudges to support their business interests, such as appealing packaging, strategic product placement, and sophisticated advertisements that link smoking to desirable traits like success, glamour, rebellion, or social acceptance.^77^^,^^78^ Similarly, the alcohol industry uses “dark nudges and sludge” to undermine scientific evidence, exploit consumers’ cognitive biases, and promote mixed messages about alcohol’s harms, particularly to the most sensitive populations such as pregnant women and their fetuses.^79^^,^^80^ Several chemical-intensive sectors, including consumer electronics and the fast- and ultra-processed food industries, use similar nudging strategies. As a consequence, global environmental and health threats are sharply rising, such as excessive plastic and e-waste generation and the prevalence of unhealthy dietary habits.^81^^,^^82^^,^^83^ The fluoropolymer industry serves as a notable example within the chemical-intensive sector for effectively using behavioral nudges to downplay the risks of Teflon exposure while exerting pressure on regulators and funding studies.^56^ The industry overemphasized the convenience, ease of cleaning, and durability of fluoropolymer-coated cookware while downplaying safety concerns related to PFAS release from these products.^84^^,^^85^ Apart from businesses, governments in several countries have employed behavioral interventions to encourage their citizens to fulfill civic duties, such as paying taxes fairly, promoting selected policies on sustainable mobility and tourism, and adopting energy-saving measures.^86^^,^^87^^,^^88^^,^^89^^,^^90^

Concerning chemicals management, employing behavioral nudging may seem like a less forceful and relatively soft approach compared to traditional regulatory interventions that directly address the production system of chemicals and chemical products. However, studies have shown that behavioral nudging can be equally effective and, in some cases, provide greater net societal benefits than traditional regulations.^86^^,^^91^ For instance, the Government of India has persistently attempted to eradicate open defecation and enhance the nation’s water, sanitation, and hygiene (WASH) status through traditional regulatory programs for over 40 years. Yet, it is only through the recently and diligently implemented behavioral interventions and shifting social norms that these WASH policies have gained substantial momentum. Specifically, they markedly reduced open defecation, promoted hand hygiene practices, and consequently contributed to curbing the transmission of waterborne and soil-transmitted diseases.^92^^,^^93^^,^^94^^,^^95^ Similarly, in recent years, researchers have begun to thoroughly explore the potential of integrating behavioral nudging into the effective implementation of traditional regulations and policies addressing climate change and conserving biodiversity, the other two segments of the triple planetary boundary crisis.^96^^,^^97^^,^^98^ Nevertheless, the integration of behavioral nudging has not been systematically recognized or implemented in the chemicals management sector until now. Given the emerging success of behavioral nudging in addressing various public and private sector ambitions and challenges, there is compelling yet currently underexplored potential in implementing such interventions in the chemical management sector.

## Next steps: Applying behavior nudging for safe and sustainable chemical consumption

Rising consumerism and economic growth have significantly boosted the global chemical industry in recent decades, particularly in the United States, the United Kingdom, EU, Japan, Australia, China, and India.^99^ The growth in consumerism and the chemical industry in these regions and countries is reflected in their substantial contributions to global material footprints, which measure the total amount of raw materials extracted to meet final consumption demands.^100^^,^^101^ Globally, material footprints have surged alarmingly: between 1990 and 2017 alone, they rose by 50%, from 8.1 to 12.2 tonnes per capita. In 2017, high-income countries had a material footprint of 26.3 tonnes per capita, surpassing upper-middle-income countries by 60% (16.9 tonnes).^100^^,^^102^ Moreover, during this period, the material footprints of high-income countries exceeded their domestic material consumption, indicating that lifestyles in the wealthiest countries are not fully met by their own resources but also heavily rely on resources extracted from poorer countries.^102^^,^^103^^,^^104^ Apart from growing consumerism, which exacerbates the extraction of resources to meet local and global demands, poorer countries often face additional challenges arising from the prevalent “throwaway” consumerism culture particularly from the affluent societies. Furthermore, these countries adopt and implement circular economy practices less effectively and holistically than developed countries, which worsens their waste management and chemical pollution issues.^105^^,^^106^ Intense consumerism has been shown to exert a heavy toll on both the environment and human health, with single-use plastic and microplastic pollution being among the most visible and well-documented chemical challenges caused by over-consumption.^107^^,^^108^^,^^109^

Achieving effective sustainable consumption to minimize material footprints (along with chemical consumption) necessitates significant efforts to shape the behavior and decision-making processes of consumers and additionally of businesses and governments. Consumers play the most important role in the “consumption system” and can be nudged toward safer and more sustainable practices through various behavioral and structural interventions (see Figure 1; Table 1).^110^ According to the European Environment Agency (EEA), the main factors influencing consumer behavior are (1) economic factors, (2) the fit between needs and offerings, (3) information, (4) social factors, and (5) preferences and beliefs.^111^ Additionally, Rare’s levers for behavior change add (6) rules and regulations and (7) emotional appeals to the list of factors described by the EEA.^112^ It is important to acknowledge that there is no scientific consensus on the precise set of factors shaping consumer behavior, and these selected factors highlighted here are based on the limited research available. Primarily, these levers of behavior change consider that the inclination toward growing consumerism is driven not only by basic needs but also by desires for novelty, status, and social comparison. However, by using tools from marketing and behavioral science, consumer perceptions and behaviors can be directed toward more sustainable and chemically safe choices.^113^ A recent study introduced a framework based on five key drivers of sustainable behavior change: social influence, habit formation, individual self, feelings and cognition, and tangibility.^114^ The study showed that understanding and leveraging these drivers, along with targeted behavior change strategies within each driver, can enable more sustainable consumer actions. Moreover, social factors, such as social norms and peer influences, often play a critical role in shaping consumption behavior, in many cases perpetuating cycles of unsustainable consumption.^115^^,^^116^

**Table 1:** A set of key actions for behavior-shaping among different target populations, groups, and innovation opportunities for the chemical industry toward improved and effective global chemicals management

Target population group	Key behavior-shaping interventions and actions	Innovation opportunities for the chemical industry
Populations that are vulnerable and sensitive to chemical exposure and resulting health consequences. • Pregnant women and their offspring • Women of childbearing age • Adolescents	Information: • Develop targeted educational and awareness programs (in accessible language) that inform these population groups about the risks of chemical exposure and practical ways to minimize it. • Provide hands-on training and workshop sessions in community centers, schools, and prenatal clinics focusing on identifying hazardous chemicals in everyday consumer products, food, and the environment. • Advocate for policies that mandate clear labeling of chemical contents in consumer products and restrict the use of proven harmful chemicals, especially in products targeting children and women. • Use social media to spread the right information about reducing chemical exposure. Incentives: • Implement programs that reward families or individuals for adopting safer practices, such as switching to non-toxic products or following safe dietary practices. Social support: • Engage community leaders and peer groups to foster a support system that reinforces safe and sustainable behavior and mutual accountability toward the consumption of chemical-intense products. • Initiate community programs for teaching skills in reading and decoding product levels, preparing natural cleaning products, household hygiene etc. • Involve community members to push for greater transparency from manufacturers. • Create community forums or workshops/webinars where experts can answer questions and provide personalized guidance on chemical exposure topics. Build safe indoor and outdoor space and infrastructure: • Establish play areas, maternity clinics, kindergartens, and schools in environmentally safe zones with modern safety protocols that minimize exposure to chemicals in the environment as well as building materials, such as carpets, flooring, paints, etc.	• Place of warning labels and visual cues on consumer and household products and food items. • Clear product labeling to illustrate chemical safety for sensitive population groups (smart labeling and digital traceability of product ingredients). • Accessible mobile applications that can scan products for harmful chemicals and/or offer safety tips for pregnant women and parents. • Reward systems from large supermarkets and grocery stores to acknowledge the sustainability efforts of consumers. • Ensure subsidies or financial support for safer alternatives to common products containing hazardous synthetic chemicals. • Manufacture household products that are chemically safe for sensitive population groups.
General public • At personal and community levels	Information: • Launch multimedia campaigns (e.g., social media, TV, print) that inform the public about the health impacts of priority toxic chemicals in everyday products and options for safer alternatives. • Conduct community workshops on sustainable living and safe and sustainable product usage to educate people on reducing their chemical footprints. • Promote programs that teach consumers to give due consideration to product labels, highlighting key chemicals risks and safe choices. • Promote mobile apps that allow users to scan product barcodes to check for harmful chemical ingredients and suggest safer alternatives. Incentives: • Offer financial incentives such as discounts, coupons, or loyalty points for purchasing chemically safe and environment-friendly products. • Provide subsidies or tax benefits for purchasing non-toxic, eco-friendly products. Social support: • Create community recognition programs that celebrate households or individuals who demonstrate significant reductions in chemical footprints. • Collaborate with influencers and trusted figures and celebrities to advocate for safer consumption choices and chemical-free lifestyles. • Organize challenges at community and personal levels (e.g., “Plastic-Free Month” or “Non-Toxic Week”) that encourage people to collectively reduce their use of products with harmful chemicals. • Highlight testimonials and success stories from people and communities who have successfully reduced their chemical footprints, emphasizing health and environmental benefits. • Host community-level workshops and provide resources on how to make safe, chemical-free alternatives for household cleaning, personal care, and beauty products. • Conduct chemical awareness days in schools, offices, etc. on benefits of using chemical-friendly options. • Establish easy-to-access programs for safe disposal of products containing harmful chemicals, reducing environmental contamination, particularly in developing countries and marginalized communities.	• Place eco-friendly and safer products at eye level or in prominent locations in grocery stores to subtly encourage their purchase. • Promote labels or shelves or notifications through store apps to remind consumers about choosing safer and essential-use chemical products. • Add stickers or icons on products that demonstrate products are low in harmful chemicals, making it easier for consumers to choose safer options. • Increase the availability of safer, low-chemical products in popular stores and marketplaces. • Develop and distribute recommended products that are verified as safe and sustainable by third-party organizations. • Promote subscription options to supply consumers with non-toxic household items and personal care products. • Develop certification programs (e.g., “Chemical-Safe Certified”) that help consumers identify safer products. • Incorporate an “eco-scoring/chem-scoring” feature into the mobile applications of popular grocery and retail chains. • Provide consumers with data-driven information on the impact of their purchase, e.g., choosing product X, you helped reduce harmful chemical use by factor Y. • Organize product swap events where consumers can exchange their high-chemical items for safer, greener options. • Set up refill stations for consumer products as well as food products to cut down on plastic waste and chemical exposure.
Farmers	Information: • Dedicated and regular workshops and seminars to educate farmers on the risks of chemical exposure, safe handling, and the long-term health impacts on themselves and society. • Programs to guide farmers to adopt organic farming. • Encourage the use of protocols that farmers can use before handling fertilizers, pesticides, and other agrochemicals. • Practical demonstration of safe and only necessary application of agrochemicals. • Provide access to resources and training for alternative pest-control methods, crop rotation, intercropping, and organic pesticides. • Provide easily accessible online training programs and resources farmers can access on mobile devices. Incentives: • Financial subsidies for adopting integrated pest management practices and switching to less toxic and more eco-friendly products. • Incentivize organic farming. • Subsidize safer agrochemicals. Social support: • Establish community-based recognition or reward programs for farmers for adopting the best safe and sustainable practices in agrochemical application and use. • Develop a network of peer leaders who can share experiences, provide mentorship, and promote the adoption of safe practices with farming communities. • Foster a sense of community by periodic events where farmers exchange their experience with safety protocols. • Disseminate data-driven success stories of other farmers who have successfully implemented safe practices, highlighting benefits to the environment and health of consumers, crop yield, and economic benefits.	• Require manufacturers to include clear safety and warning labels on all agrochemical products, making it easier for farmers to understand the risks and necessary precautions. • Require compliance with regulations, mandating proper labeling and only essential use of chemicals in agrochemicals.
Workers across different chemical-intense industries and sectors^a^	Information: • Conduct tailored training programs for each sector that focus on specific chemicals, safe chemical handling, managing spills, responding to emergencies, risks, and protective measures relevant to the jobs. For instance: on handling hazardous chemicals and their wastes, recognizing early signs of exposure; safe handling and disposal of lead, asbestos, and other construction chemicals, etc. • Provide safety reminders, visible personal protective equipment stations, and color-coded labels for chemicals and tools to quickly identify hazard levels and relevant protective measures. • Create sector-specific digital learning modules that workers can access on mobile devices. • Implement mandatory certification programs for workers handling chemicals to ensure baseline knowledge and safe practices. Incentives: • Reward programs for teams, individuals, and brands. • Provide subsidized high-quality PPE to ensure workers’ safety, particularly for workers in informal sectors, e.g., waste pickers and recyclers in developing countries. • Incentivize the use of safer chemical substitutions at workplaces. Social support: • Leverage peer-to-peer education where experienced workers teach and reinforce safe practices among their colleagues. • Create online or in-person platforms where workers can share tips, experiences, and solutions related to chemical safety at workplaces. • Implement an anonymous reporting system that allows workers to report unsafe practices or exposure incidents. • Raise awareness within the broader community to support behavior change among informal-sector workers.	• Develop and promote the use of mobile apps or sensors that can track exposure levels and send warnings upon exceedance of safety thresholds. • Implement measures for continued indoor environment monitoring and biomonitoring of workers to keep check on chemical exposure levels.

a Some of the relevant industrial sectors where chemical exposure is significant: (1) Chemical manufacturing, (2) Construction and demolition, (3) Cleaning and janitorial staff, (4) Beauty and personal care industry workers, (5) Transportation and maintenance workers, (6) Mining and oil & gas extraction workers, (7) Textile and apparel workers, and (8) Informal sector workers, e.g., waste pickers and recyclers.

At the individual level, shaping a safer and more sustainable “consumption system” can be achieved by using various levers of behavior change. For instance, consumers can be provided with clear information about whether a product may expose them (or the environment) to harmful chemicals and to what extent. This can be done by making safe and sustainable options more visible and appealing, e.g., through clear and prominent “chemical safety labeling or rating”.^117^^,^^118^ This can be complemented by choice-editing techniques, which involve offering the most chemically safe and sustainable options as the default choices,^119^ for example, by placing these products at eye levels where they are more likely to be noticed and purchased. Such nudging interventions should particularly target vulnerable population groups, such as pregnant women, women of childbearing age, caregivers of newborns, and small children (given that children up to age 3 are strongly sensitive to the effects of hazardous chemical exposure).^120^ Specific and focused chemical risk communication is crucial to reach these vulnerable population groups. Influential figures, including social and religious leaders, celebrities, and other public figures, can play an important role as multipliers to reach vulnerable groups as well as the general public. By leveraging their platforms, these influential figures can amplify awareness and promote safer and more sustainable consumption behaviors, especially among high-risk populations.^121^ Research has shown that programs aimed at changing behavior related to maternity and childbirth have a positive impact on reducing maternal and neonatal mortality, as well as the prevalence of malnutrition, in developing countries.^122^ Important levers for these critical changes included the introduction of incentives and the combination of individual and group counseling (through promoting social and cultural changes).^122^^,^^123^

From a chemicals management perspective, these programs need to include focused packages to minimize maternal and neonatal exposures to toxic chemicals, especially for exposure through diet, consumer products, cosmetics, the indoor environment, and baby products. Behavioral interventions, using different combinations of relevant levers such as easily accessible and understandable “information” and “social and economic factors”, can help the vulnerable groups make informed decisions for choosing toxic-chemical free and holistically (covering different toxic endpoints) safe options (of dietary, consumer, and lifestyle choices) according to the contemporary scientific understanding.^124^ Furthermore, in marginalized communities (including in developing countries), information on chemical safety is not adequately accessible. In such cases, leveraging social norms and peer influences can foster positive behavioral shaping by highlighting safe and sustainable consumption practices adopted by others, and providing real-time feedback on consumers’ actions.^124^^,^^125^^,^^126^ Importantly, combining multiple levers to address various cognitive biases has been shown to increase the overall effectiveness of behavioral interventions.^110^^,^^127^

By better understanding and influencing consumer decision-making processes and biases, it is possible to initiate transitions toward a society that designs and consumes chemicals in alignment with long-term SDGs. Furthermore, fostering consumer demand for chemically safe products can accelerate the efforts of first movers in the chemical industry, enabling them to overcome the substantial investment requirements and market uncertainties that currently hinder their transition to safer and more sustainable practices. For example, Eastman 168, a non-phthalate plasticizer, with low hazard concerns, has been a significant driver of its producer’s financial growth, positioning the company as a major player in the global plasticizer market.^128^ Another example is Fenix Outdoor’s proactive elimination of PFASs from its products. This has offered the company a competitive advantage in the market, especially in times when regulatory pressure concerning PFASs is mounting in the EU and other developed regions. This early adoption of PFAS free production practices not only mitigates regulatory risks but also enhances its brand reputation and appeals to a growing segment of eco-conscious consumers.^129^

## Key actors and their roles for enabling behavior shaping for safe and sustainable chemical consumption

Based on the experience of implementing behavior interventions to address other environmental (and social) challenges as well as challenges specific to chemical pollution (see Box 1),^96^^,^^98^^,^^103^^,^^114^^,^^130^^,^^131^ it is essential to define who is responsible for implementing different components of behavior-shaping strategies for safe and sustainable chemical consumption. This clarity is not only fundamental to the effectiveness of these strategies but also required for enhancing coordination among key actors, each of whom has a unique set of capacities and responsibilities.

Box 1 **Examples of key actors’ vital roles in shaping consumer behavior for the protection of the environment and human health**

**Civil Society Organizations**: EcoWaste Coalition, a local NGO in Southeast Asia, uses various nudging interventions to educate families on mercury- and lead-free consumer products including cosmetics and paints.^132^^,^^133^

**Government and regulatory agencies**: The UK government’s behavioral insights team’s (BIT) advice led to widespread availability of e-cigarettes throughout the country which turned out to be a more effective and dominant route to quitting smoking than other rival methods.^134^

**Academic and policy think-tanks**: The Forever Pollution Project, which has run for over two years since 2023 and involved cross-border collaboration among journalists, academics, and lawyers, is an example of how scientific findings can be translated to policymaking.^135^^,^^136^ The project played a pivotal role in elevating the issue of PFAS pollution to the top regulatory level within the EU and partially globally. Its efforts provided important support (in terms of information, outreach, and political agenda setting) for the EU’s proposed “universal restriction” on PFASs under the EU REACH regulation.^135^

**Industry**: IKEA has been phasing out PFASs from its products over the last 15 years with an ambition to have all IKEA cookware and bakeware ranges PFAS-free by 2026. IKEA’s efforts contribute to a growing marketing trend, building a corporate momentum behind the phase-out of PFASs. It also reflects that “PFAS-free” is a good marketing. Along with IKEA, more than 120 consumer companies have joined ChemSec’s PFAS Movement, officially supporting a comprehensive PFAS ban and committing to phasing-out PFASs from their products.^137^^,^^138^

### Key actors

A key primary actor and change facilitator in this context is CSOs.^139^ They are often in close contact with local communities, possessing a deep understanding of local needs and knowledge, cultural boundaries, and community trust. This gives them an advantage over other stakeholders in co-designing and tailoring behavior-shaping interventions to address specific societal barriers. Furthermore, CSOs serve as translators between policy frameworks (and academic findings) and their on-ground implementation, helping to ensure that the interventions promoting safe chemical consumption are not only technically sound but also socially acceptable.^140^ In regions where the functioning of formal policy institutions is weak or mistrusted, CSOs can not only informally reinforce behavior-shaping strategies through socially acceptable mechanisms and community norms but also help to protect consumer and labor rights.^141^ Furthermore, CSOs can act as stewards of behavior-shaping programs in the long-run, sustaining and monitoring momentum even after donor-funded or government-led interventions have ended.

Importantly, the shaping of consumer behavior toward safer chemical consumption is not driven by awareness campaigns alone, but also dependent on the presence of a robust civil society and credible enforcement authorities. Civil society actors with the necessary expertise can advocate transparency, mobilize communities, and pressure industry to adopt safer and only necessary chemical use and production practices. Nevertheless, it has to go hand in hand with an effective enforcement of existing chemical regulations to prevent unsafe or illegal activities, particularly in regions where informal markets and regulatory evasion are widespread. In lower-capacity contexts, building enforcement infrastructure and addressing illegal practices must be part of any comprehensive behavior change strategy. This calls for integrated approaches that combine community engagement, public-institutional strengthening, and international cooperation to ensure both industry accountability and consumer protection.

Government and regulatory agencies are important actors in implementing actions shaping consumer behavior for safer and more sustainable chemical consumption.^141^ Foremost, they provide the regulatory foundation and financial infrastructure that underpin many behavioral strategies. Through legislation, enforcement, and the creation of enabling environments, governments can embed behavior-shaping interventions into broader policy architectures. The upcoming “Right to Repair” directive in the EU is an example of empowering consumers by giving them more possibilities of repairing their products and services, and thus further reducing or avoiding the possible chemical pollution as a result of just discarding the product.^142^ In ideal scenarios, regulatory agencies can directly implement and enforce behaviorally informed policies, such as by mandating chemical safety labeling on food and consumer products, and occupational safety nudges. Such policy interventions are especially relevant in countries where industry lobbying is strong and compromises consumer health and safety as well as with environmental management.^143^

While much of the literature on behavioral interventions to nudge consumers for safer choices is rooted in experiences from more advanced regulatory environments, many global contexts are marked by informal practices and fragmented enforcement as well.^98^^,^^144^ In developing economies, limited institutional trust and weak regulatory infrastructure create significant barriers to the successful deployment of nudges and other behavior-shaping tools.^145^ In these settings, decentralized governance approaches become a way forward. For example, behavioral strategies may be independently implemented by the local (instead of central) government agencies in partnerships with CSOs, industries, religious leaders, traditional authorities, or community influencers who hold high social legitimacy.^146^ Similarly, participatory approaches, such as community monitoring, local pacts, or peer-to-peer enforcement, can help extend the reach of interventions beyond formal regulatory channels. In such cases, digital tools may offer additional leverage, enabling the dissemination of behavior change messages and the collection of feedback even in hard-to-reach or under-resourced regions.^147^

Industry represents another critical actor of the behavior-shaping system in the context of marketing and promoting chemically safe products and can exert substantial influence over consumers. Their operations frequently shape the environment in which consumption behaviors form, be it through product design, marketing practices, supply chain management, or workplace norms. Importantly, many of the key actions for behavior-shaping proposed in this perspective (see Table 1) can align with business interests, such as marketing household and consumer products that are sustainable and chemically safe for sensitive population groups, ultimately offering new opportunities for innovation. By ensuring safe chemical production and use, industry may benefit from improved safety outcomes, reduced liability, enhanced brand reputation, and increased customer loyalty.^148^^,^^149^ Framing behavioral innovation as a driver of market differentiation and operational efficiency, not just for regulatory compliance, can help secure businesses stronger buy-in from private sector partners and the broader supply chain. Trade associations, meanwhile, can serve as multipliers, diffusing best practices across firms and facilitating voluntary codes of conduct that internalize behavior-change principles.^150^

Academic institutions and think tanks play a crucial role in the design, implementation, and scaling of behavior-shaping interventions. Here, it is important to acknowledge that in the field of environmental health sciences, the role of researchers goes beyond the conventional responsibilities typically observed in other academic disciplines. Their primary contribution in this interdisciplinary academic domain not only lies in generating reliable and accessible information that has been evaluated independently, as well as evidence-based insights that inform policy and practice, but also in participating in science-policy negotiations and channeling the information to other relevant actors.^151^^,^^152^ Through their empirical research, experimental trials, and longitudinal studies, they may further help to identify which behavioral strategies work for global and local chemical management, for whom, and under what conditions. Beyond producing knowledge, academic institutions also serve as trusted evaluators, offering independent assessments of these interventions’ outcomes that can guide further refinement and replication in different socioeconomic and cultural settings. Additionally, they can contribute to capacity building by training practitioners, policymakers, and industries and CSO representatives by ethically applying behavioral science principles and methods toward safer chemical consumption. Beyond academic institutions, think tanks serve as intermediaries, translating academic findings into actionable guidance to governments, CSOs, and industry stakeholders for shaping consumer behavior. These actors help to bridge the often-wide gap between theoretical knowledge and practical application, ensuring that interventions are both methodologically sound and contextually relevant. Furthermore, academic institutions and think tanks often have the convening power to bring together diverse stakeholders across sectors, thereby facilitating collaboration and fostering shared understanding around complex behavioral challenges. In this way, they not only produce knowledge but also actively shape the ecosystems that support effective behavior change.

### Key actors’ roles and essential prerequisites for successful shaping of consumer behavior

**Figure 2:**
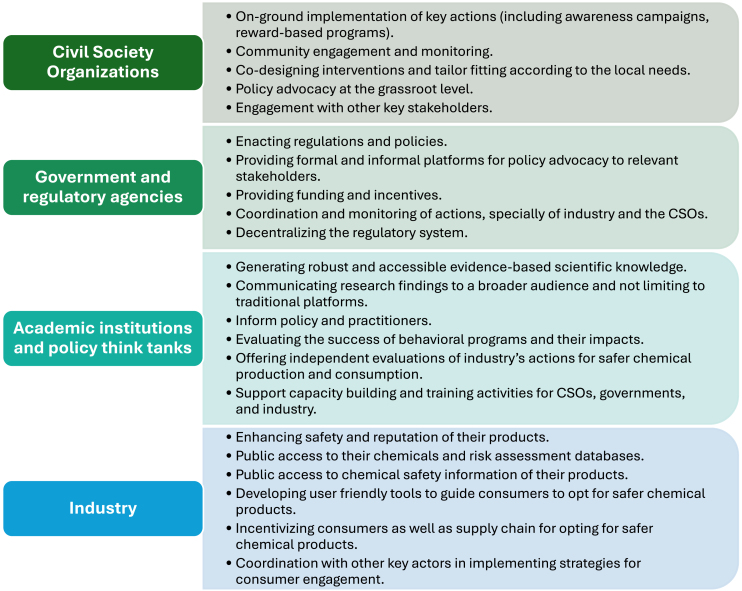
A summary of important actions for influencing consumer behavior, grouped by relevant actors

Success of the behavior shaping actions highlighted in Table 1 and Figure 2 depends not only on how well-designed these actions are but also on clearly defined allocations of responsibilities among the key actors. Some of these responsibilities are discussed hereafter.

Government plays a major role in formulating and validating policies, meanwhile also coordinating and enabling roles across other actors and their respective components. The role of governments goes beyond “just” providing the regulatory foundation and financial infrastructures. For instance, governmnts are responsible for validating educational content and the use of various formal and informal platforms. They are also responsible for ensuring that the outreach of these activities reaches all demographic groups. Toward incentivizing these actions, governments’ role is foreseen in allocating appropriate funding (e.g., through environmental financing) for sustaining the reward system, be it for the consumers or the industry for safer chemical consumption and production, respectively.^153^ These incentives could be in the form of tax benefits, product subsidies, or procurement advantages for chemically safer products.^154^

CSOs implement these actions and activities at the grassroot level by engaging the community using culturally appropriate formats to channel the right information about the chemicals’ risks and safer choices.^154^ CSOs are also at the forefront in implementing reward-based programs, including safer household practices, toxic-chemical-free product purchases, and organic farming adoption. Moreover, their proximity to consumers allows them to monitor public engagement and ensure fairness in access to incentives and benefits. Their role is also foreseen in fostering social support to these key actions through engaging community leaders, organizing peer-led networks, and running social campaigns (both through online and offline sources).

Industry is seen as both a source of chemical risk and a critical partner in solutions. In channeling the appropriate information on chemical safety, industry has an important role to play, for instance, by committing to transparent labeling of chemical contents in consumer products. Large supermarkets and online retailers can also develop user-friendly tools that inform consumers about safe usage, safety standards, and safer alternatives. Here, their role also extends further to ethical marketing, ensuring that their products, especially those used by vulnerable populations, meet chemical safety standards. In terms of incentives, industries can directly offer consumers rewards for opting for safer products, this may include loyalty programs or discounts. Internally, they may also implement strategies to reward programs that drive safer chemical consumption across their supply chains. Through their CSR activities, industry can go beyond the traditional environmental campaigns to support more sophisticated programs to promote a low-chemical risk lifestyle in society.

The role of academic institutions and think tanks, as knowledge providers, is to generate scientific evidence-based knowledge tailored to the local societal needs, support translating their findings into educational and awareness resources that are accessible to both policy makers as well as CSOs and different sectors of the general public including farmers and industry workers. As evaluators, their role is to assess the impacts of various behavior shaping programs and community-led solutions, and guide policy makers and CSOs through evidence-based evaluations.

Building on the need to shape safe and sustainable chemical consumption through targeted behavioral interventions, researchers can play a pivotal role in addressing the challenges of unsustainable practices at industry and governmental levels while synthesizing guidance to overcome these issues. Collaborative exploration with industry and government can help identify pathways for balanced economic growth in the chemical sector that prioritizes consumer and environmental safety. This includes investigating mechanisms to counteract biases such as status-quo bias and risk aversion in corporate decision-making.^130^^,^^155^ Research should also focus on the potential of alternative business models, to safeguard sustainability-focused companies in the chemical sector from shareholder pressures to prioritize short-term profits. Furthermore, regulatory interventions, including those mandatory for chemical safety and sustainability reporting, health and environmental profit-and-cost statements, and chemical taxation, warrant further investigation to evaluate their effectiveness in driving systemic transformations in this sector. Equally critical is examining how developed and developing economies can adopt alternative metrics for measuring societal progress, such as the Genuine Progress Indicator (GPI) or Gross National Happiness (GNH), to promote safe and sustainable consumption of chemicals and reduce reliance on conventional growth measures.^156^ These research efforts will contribute toward synthesizing actionable strategies to better integrate consumer, industry, and government roles in achieving effective global chemicals management.

Practitioners, meanwhile, can complement these research-driven contributions by implementing measures to promote safe chemical production and sustainable consumption practices. For instance, emphasizing the risk of stranded assets and financial losses related to health-costs (both at the societal and occupational levels) can encourage a shift toward safer and responsible business strategies.^157^ Practitioners can focus on ensuring the implementation of detailed chemical safety and sustainability reports, which enhance accountability and provide a framework for aligning production processes with environmental goals. They can leverage economic tools, such as tax incentives and penalties to encourage the industry to adopt safer chemical alternatives while setting regional benchmarks. Additionally, practitioners can foster collaboration across sectors by establishing public-private partnerships to support investments in cleaner and safer technologies and designing consumer engagement strategies to emphasize the benefits of safe products and sustainable consumption. By addressing these areas, practitioners can create an enabling environment among consumers that shifts market dynamics toward safer chemical consumption practices, contributing to the broader transition to a chemically sustainable society.

While behavior-shaping interventions hold significant promises for promoting safer chemical consumption, their effectiveness depends on consumers having access to reliable and comprehensible information. Independent evaluation and inventorization of hazard and risk data is essential to ensure credibility and prevent misinformation about chemicals and chemical-containing products.^158^^,^^159^ At present, especially in developing countries, much of this information is generated by industry to meet regulatory requirements, which, while valuable, may not always be framed in ways that are accessible, understandable, or actionable for the general public.^154^ Bridging these gaps in reliable data accessibility requires investment in publicly available databases, consumer labeling schemes, and digital tools that translate technical information into meaningful guidance for real-life exposure scenarios. Furthermore, channeling this knowledge to the general public requires greater participation of CSOs as well as of academic institutions and think tanks. At the same time, it is important to recognize that behavior shaping, as part of industry’s and the private sector’s nudging programs, is already widely used, often promoting sales and resist regulations. From this point of view, advancing a public-interest use of behavior-shaping strategies demands safeguards, transparency, and oversight to ensure that such approaches genuinely support health, safety, and sustainability goals.

Ultimately, the success of behavior-shaping interventions for safer chemical consumption depends not only on their design but also on the ecosystem in which they are embedded. A strong and well-organized civil society, credible and capable public institutions, motivated and incentivized industry actors, and culturally feasible implementation are all essential components for the success of these interventions. Acknowledging these interdependencies and reflecting them in the design of behavior change policies can significantly enhance their long-term effectiveness, particularly in complex and diverse regional contexts. In this direction, future research could explore the scope of behavior shaping interventions in diverse socioeconomic systems, the dynamics of ethical nudging within specific branches of chemical industry, and the role of media (including social networking) in both shaping public awareness and influencing how issues of chemical pollution are understood, debated, and prioritized within experts, policymakers, or industry community. Furthermore, research could focus on assessing key factors contributing to successful nudging interventions across diverse spectrums of society and chemical industry.

## Acknowledgments

B.M.S. acknowledges support from the OP JAK MSCA-CZ, OP JAC-Project MSCAfellow5_MUNI (No. CZ.02.01.01/00/22_010/0003229)–co-funded by the 10.13039/501100000780European Union (EU). B.M.S. and M.S. acknowledge the research infrastructure RECETOX (LM2023069) financed by the Ministry of Education, Youth and Sports of the Czech Republic for supportive background. This work was supported by the European Union’s 10.13039/501100007601Horizon 2020 research and innovation program under grant agreement no 857560 (CETOCOEN Excellence). This publication reflects only the author’s view, and the European Commission is not responsible for any use that may be made of the information it contains.

The authors thank Prof. Leonardo Trasande, MD, (NYU Langone Medical Center, New York City (NYUMC)) and Dr. Anežka Sharma (RECETOX, Masaryk University) for their valuable feedback on the manuscript.

## Author contributions

B.M.S. conceptualized this perspective with input from M.S., L.Z., J.M., J.M.B., T.A.B., and P.A. B.M.S. prepared the preliminary draft. All authors reviewed the manuscript, provided feedback, and approved the final version. All authors were responsible for the decision to submit the manuscript for publication.

## Declaration of interests

All authors declare no competing interests.
